# In Vitro Assays in Severe Cutaneous Adverse Drug Reactions: Are They Still Research Tools or Diagnostic Tests Already?

**DOI:** 10.3390/ijms18081737

**Published:** 2017-08-10

**Authors:** Grzegorz Porebski

**Affiliations:** Department of Clinical and Environmental Allergology, Jagiellonian University Medical College, Sniadeckich 10, 31-531 Krakow, Poland; porebski@mp.pl; Tel.: +48-12-423-11-22

**Keywords:** drug hypersensitivity, diagnosis, in vitro, T-cells, SJS, DRESS, AGEP

## Abstract

Severe cutaneous adverse drug reactions (SCARs) represent life-threatening medical conditions and an appropriate causative diagnosis of these conditions is of the highest importance. Existing in vivo diagnostic methods are risky or are just contraindicated in these patients. Therefore, in vitro tests take on greater significance. In this survey, the studies on in vitro assays in SCARs were identified with a defined searching strategy and strict eligibility criteria. Different methods in the particular clinical manifestations and the groups of drugs were compared in respect to the diagnostic parameters obtained. The lymphocyte transformation test and IFNg-ELISpot (Interferon γ-Enzyme-linked immunospot assay) appeared to have the best evidence currently available. Further diagnostic assays, which are based mostly on distinct mechanisms of SCARs, may outdo previous assays but they still need confirmation in a larger group of patients and in more research centers. Data from pediatric populations and acute generalized exanthematous pustulosis (AGEP) patients are scarce. Some technical issues, limitations, and modifications of routine laboratory methods are also discussed.

## 1. Introduction

Adverse drug reactions (ADRs) are of significant concern for clinicians and patients. Although effective and potent drugs are important advances in modern medicine, they are also a major cause of iatrogenic diseases. Hence, ADRs represent an actual public health problem [1]. The classification proposed by Rawlins and Thompson may serve as an initial approach to ADRs [2]. They divided ADRs into type A and type B reactions. Type A reactions, which represent 75–80% of all ADRs, are dose-dependent, predictable, related to drug pharmacology, and occur in nearly all individuals, whereas type B reactions, which account for the remaining 20–25% of ADRs, are considered to be unpredictable, dose-independent, typically unrelated to the pharmacological action of a drug, and occur only in susceptible patients [3,4]. Type B reactions are also called idiosyncratic drug reactions [4]. They include immune-mediated drug hypersensitivity reactions (real drug allergies) and nonallergic hypersensitivity (also called pseudoallergy), which are not immune mediated, e.g., an alteration in arachidonate metabolism (hypersensitivity to nonsteroidal anti-inflammatory drugs) or a bradykinin accumulation due to the intake of angiotensin-converting enzyme inhibitors [1].

Immune-mediated drug hypersensitivity reactions (DHRs) are classified into four types (I–IV) described by Coombs and Gell [5]. The most common are IgE- and T-cell-mediated reactions, corresponding to type I and type IV, respectively. Type II reactions are based on IgG and IgM mediated cytotoxic mechanisms. Type III reactions are mediated through an immune complement complex. Different effector functions of drug-activated T cells led to a subclassification of type IV into type IVa–IVd [6]. Type IVa and IVb are driven by T-helper 1 cytokines (IFNg, TNFa) and T-helper 2 cytokines (IL-4, IL-5, IL-13), respectively. Eczemas are typical manifestations of type IVa DHRs, whereas maculopapular exanthema and drug rash with eosinophilia and systemic symptoms (DRESS) represent type IVb reactions. Type IVc reactions rely on effector cytotoxic T cells releasing cytokines such as granzymes, perforin, and granulysins. These cytotoxic mechanisms predominate in drug-induced bullous skin reactions such as Stevens–Johnson syndrome and toxic epidermal necrolysis but can be present in other delayed DHRs. Type IVd reactions correspond to IL-8-dependent sterile neutrophilic inflammation, which is involved in acute generalized exanthematous pustulosis [3].

Coombs and Gell classes, however useful, do not completely cover our current understanding of the underlying pathophysiology of DHRs. Modifications of nomenclature and classification are still debated [1,4,7]. The classical explanation for DHRs states that a drug acts as a hapten and binds covalently to proteins. This process results in the alternation of the protein structure and the generation of new antigens (hapten–protein complex) [8]. Some drugs are prohaptens and require metabolic activation (e.g., sulfonamides) to gain the ability to bind irreversibly to protein [1]. Accumulation of reactive nucleophiles to metabolites may also provide important maturation signals, often referred to as “danger signals” [9], which play a role in the activation of the innate immune system and in the development of an effective immune response. A drug and, possibly its reactive metabolite, can also directly and non-covalently bind to immune receptors such as HLA or TCR proteins [10,11], then activate T cells and stimulate a specific response (pharmacological interaction with immune receptor, known as p-i concept) or promote an exchange of peptides embedded in an antigen-binding site of HLA. The latter process may also induce severe DHRs [12].

Classification of DHRs is also challenging from a clinical point of view because of the huge variety of clinical presentations and their combinations. Moreover, symptoms of DHRs vary in respect to chronology and severity. Immediate reactions occur within 1–6 h, typically ≤1 h, after a culprit drug administration [1,4]. Apart from an anaphylactic shock, they manifest, for instance, as urticaria, angioedema, rhinitis, bronchospasm, nausea, vomiting, or diarrhea. Nonimmediate reactions require more time for the symptoms to appear and are either intermediate (5–14 days) or delayed (2–7 weeks) [13]. The skin as well as the liver and blood cells, are the most commonly affected. Symptoms range from mild maculopapular or morbilliform exanthemas, through clinical signs related to organ involvement (e.g., hepatitis, nephritis) and blood dyscrasias (e.g., thrombocytopenia, eosinophilia), to severe cutaneous adverse drug reactions (SCARs), such as Stevens–Johnson syndrome/toxic epidermal necrolysis (SJS/TEN), DRESS, and acute generalized exanthematous pustulosis (AGEP). SCARs represent life-threatening medical conditions and an appropriate causative diagnosis is of the highest importance, especially for patients receiving several drugs simultaneously.

The complete DHRs workup is based on clinical history and, if possible, on standardized skin tests, in vitro biological tests, and drug provocation tests. Existing in vivo diagnostic methods have significant limitations for SCARs. Drug provocation tests, which are considered to be the gold standard for the identification of the culprit drug, are absolutely contraindicated for severe life-threatening DHRs. Skin patch tests demonstrate low sensitivity in SJS/TEN [14] and may produce flare up reactions, especially in DRESS [15]. Moreover, many patients are afraid of re-exposition to suspected drugs. Therefore, causative diagnosis is based on the estimation of the probability of a causal relationship between a taken drug and symptoms. Additional diagnostic tests, such as in vitro tests, may bring this probability closer to the decision threshold (whether or not a patient can take a tested drug in the future). A number of studies, employing in vitro methods, revealed immunological mechanisms (mediators and effector cells) involved in the SCARs. Laboratory techniques, such as flow cytomery, enzyme-linked immunospot (ELISpot), or ELISA, may grasp distinct parameters of a drug-specific T cells response (e.g., proliferation, surface markers expression, cytokines release) [3] but the translation of these findings into diagnostic tests is still in progress. There is a gap between the objectives of researchers and the expectations of clinicians awaiting practical diagnostic tools.

So far, the reviews in this field have been mostly based on experts’ opinions and there are still many open questions such as the optimal time for testing, the best in vitro read-out system to detect drug-induced response, and the dependence of results on particular clinical manifestations of drug hypersensitivity. We focused on the distinct manifestations of drug hypersensitivity (SJS/TEN, DRESS, and AGEP) and identified in a systemic way, as described in detail in “Section 4”, the publications dedicated to in vitro assays in these types of reactions. Next, we compared the diagnostic parameters of the methods applied in the identified publications. As a specific “diagnostic method”, we considered a read-out system and a given endpoint together. We also analyzed whether the results of particular methods depend on the tested drug or group of drugs.

## 2. Results

### 2.1. General

Only nine out of 29 studies included in the analysis were dedicated exclusively to SCARs, namely to DRESS [16,17,18,19], SJS/TEN [20,21], or both SJS/TEN and DRESS [22,23,24]. The other 20 studies were conducted on patients with different drug hypersensitivity reactions (DHRs). Data concerning SCARs patients were extracted from these studies.

Altogether, 259 SCARs patients were tested 398 times with different methods. The control groups consisted of 341 individuals in total. The mean time span between the onset of symptoms and testing was 4.98 months, with a range from 1 day [23] to 10 years [25]. Children were tested in one study [26], children and adults in three studies [27,28,29]. The age of tested individuals was not specified in one study [16]. The other studies were conducted on adults. In vitro assays were applied as diagnostic tests in most of the identified articles, however, in a few studies they served strictly as a research tool.

Clinical history served as a main reference for those studied in vitro tests. A clinical drug causality assessment was carried out using the RegiSCAR criteria [22,23,24,30], the ALDEN (an algorithm for drug causality in SJS/TEN) algorithm [20,22,23], classification of Nyfeler and Pichler [27,31], the Naranjo algorithm [22,32], the Spanish Pharmacovigilance System [18], and the criteria of Karch and Lasagna [33]. Otherwise, it was based on a suggestive history, a typical clinical picture, and an exclusion of alternative causes.

### 2.2. Drug Rash with Eosinophilia and Systemic Symptoms (DRESS)

A total of 138 DRESS patients were investigated in 20 out of 29 of the analyzed studies (Table 1). The in vitro methods (presented as “specific response marker/technique”) most often applied to detect culprit drugs were: peripheral blood mononuclear cells (PBMC) proliferation/lymphocyte transformation tests (LTT), IFNg releasing cells/ELISpot assay, IL-4 releasing cells/ELISpot, and granulysin concentration in cell culture supernatant/ELISA. Sensitivities and specificities achieved with these methods are shown in Table 2. The drugs most often tested were aromatic antiepileptics (in 29 patients) and allopurinol/oxpurinol (in 28 patients) (Table 1). The results for particular drugs and their corresponding methods are shown in Table 3.

### 2.3. Stevens–Johnson Syndrome/Toxic Epidermal Necrolysis (SJS/TEN)

A total of 115 SJS patients were investigated in 16 out of 29 of the analyzed studies (Table 4). The in vitro methods (presented as “specific response marker/technique”) most often applied to detect culprit drugs were: PBMC proliferation/LTT, IFNg releasing cells/ELISpot, IL-4 releasing cells/ELISpot and granulysin concentration in cell culture supernatant/ELISA, granulysin (Grl) in CD3^+^CD4^+^/flow cytometry, granzyme B (GrB) releasing cells/ELISpot, and INFg concentration in cell culture supernatant/cytokine beads array. The sensitivities and specificities achieved with these methods are shown in Table 5. The drugs most often tested were aromatic antiepileptics (in 25 patients) and allopurinol/oxpurinol (in 20 patients) (Table 4). The results for particular drugs and their corresponding methods are shown in Table 6.

### 2.4. Acute Generalized Exanthematous Pustulosis (AGEP)

Six AGEP patients were investigated in three out of 29 analyzed studies (Table 7). Results for in vitro methods applied in these studies as well as the tested drugs are shown in Table 7.

### 2.5. Modifications

Some modifications of routine methods were evaluated in a few studies. IFNg ELISpot, which was performed using PBMCs stimulated with anti-cluster of differentiation (CD)-3/CD28 antibody-coated microbeads and IL-2 for seven days before exposure to the culprit drugs, was far more sensitive than the conventional drug-induced IFNg ELISpot or LTT [32]. Removal of Treg/CD25^hi^ cells, which have a suppressive effect on drug-induced proliferation, increased LTT sensitivity without affecting its specificity [34]. The presence anti-programmed death ligand 1 antibody (anti-PD-L1) during PBMC incubation increased sensitivity of IFNg-ELISpot assay in DRESS patients [23]. The combined addition of anti-CTLA4 and anti-PDL1 antibodies to PBMC cultures increased drug-induced proliferation in SJS/TEN and DRESS patients [24]. After the pre-incubation of PBMCs from SJS/TEN patients with IL-7/IL-15, the enhancement of the drug-specific response, measured by granzyme B and granulysin release, was inconsistent [20].

### 2.6. Additional Studies

Six studies, which provide valuable data, were not included to the analysis because they did not fulfill eligibility criteria. PBMCs, which were sampled from SCARs patients, released after stimulation with culprit drugs, a panel of Th1 and Th2 cytokines (IL-2, IFNg, IL-4, IL-5, and IL-13) detectable with ELISpot assay [35]. In SJS/TEN patients, the sFasL levels were specifically increased in the supernatant of PBMCs cultured with culprit drugs [36]. Drug-specific T cells from patients with AGEP produce significantly more IL-8 in comparison to drug-specific T cells from patients with other types of drug-induced exanthemas [37]. Measurement with ELISpot assay of drug-specific granzyme B release by PBMC from SJS/TEN and DRESS patients allowed authors to identify the offending agents [38]. LTT-positive patients with SJS/TEN, DRESS, and AGEP showed a significant CD69 up-regulation on T cells after 48 h of stimulation with the culprit drugs [39]. Among the 17 cytokines/chemokines analyzed, IL-2, IL-13, and IFNg secretion in response to sulphonamides was significantly increased in DRESS and SJS/TEN patients [40].

## 3. Discussion

### 3.1. General Considerations

The number of studies exclusively dedicated to a practical approach to in vitro diagnosis of SCARs that also fulfill obvious and relatively simple eligibility criteria is low. However, by extracting data from other studies on different DHR including SCARs, we could collect results of different diagnostic laboratory methods from over 250 SCARs patients and from even more control individuals. The mean time span between the onset of SCARs symptoms and testing (about 5 months) was reasonable, however, it remained within a wide range. We did not analyze this issue in detail because, in our opinion, there was not enough data to draw a conclusion regarding the optimal timing for testing after SCARs. The pediatric population is to a very small extent represented in the analyzed studies.

### 3.2. Assays in Particular Severe Cutaneous Adverse Drug Reactions (SCARs)

LTT has been used for detecting culprit drugs in drug hypersensitivity reactions since the 1970s [41]. Also, nowadays, this is the most often used test in DHRs, as our findings have shown (Table 1 and Table 4). LTT also served as a reference assay in studies evaluating the other methods [35,36,38,39,40]. In the studies that we identified, LTT showed a high specificity (98–99%), whereas sensitivity was considerably low for SJS/TEN patients (37%) (Table 5) but higher for DRESS patients (67%) (Table 2). Regarding the sensitivities of the particular groups of tested drugs, we did not find substantial differences between DRESS patients (sensitivities were within the range 73–83% and in the one study with piperacillin—100%) (Table 3). In SJS/TEN patients, we found the values of sensitivities from 20% (allopurinol/oxpurinol and sulphonamides) up to 48% (aromatic antiepileptic drugs) (Table 6).

The second most often used test identified with our search strategy was IFNg-ELISpot. In comparison to LTT, it was found to have a similar sensitivity and specificity in DRESS, but a, clearly, higher sensitivity (71%) in SJS/TEN (Table 2 and Table 5). Regarding the sensitivities in the particular groups of tested drugs, IFNg-ELISpot appeared to have sensitivities from 42% (abacavir) to 64% (allopurinol/oxpurinol) in DRESS, and up to 67% (allopurinol/oxpurinol) in SJS/TEN (Table 3 and Table 6).

The other methods applied for the detection of a culprit drug in SJS/TEN or DRESS were investigated in a very limited number of studies. These methods primarily follow distinct effector mechanisms of SJS/TEN or DRESS (for instance granulysin or granzyme B release) and seem promising. However, it is difficult to draw convincing conclusions from their results because they were conducted with a single drug only (Grl-ELISA, sensitivity 86% in SJS/TEN) [22] or in a single center (Grl in CD3^+^CD4^+^, sensitivity 56% in SJS/TEN; IL-4-ELISpot, sensitivity 90% in DRESS) [20,26,42] and, until now, an overall number of tested individuals has generally been small.

There are more different in vitro methods, which have been investigated in SJS/TEN (Table 4) rather than in DRESS (Table 1) because SJS/TEN is more demanding in respect to a causal diagnosis. Data on in vitro diagnoses of AGEP are very limited (Table 7) but LTT seems to be efficient in this indication. Some valuable studies have been missed, for instance because data for a particular type of clinical manifestation (DRESS, SJS/TEN, AGEP) could not be extracted from pooled results [43]. Patch tests, which can improve the in vitro test results, seem to be of value for DRESS and AGEP, with sensitivities of 64% and 58%, respectively [14]. In the same study, the sensitivity of patch tests in SJS/TEN patients was only 24% [14]. However, it can reach up to 62.5% in some selected populations (e.g., carbamazepine-induced SJS/TEN HLA-B*1502+ patients) [44].

### 3.3. Technical Issues and Limitations

In opposition to laboratory tests for immediate DHR (specific IgE, basophil activation test), in vitro diagnostic assays in delayed DHRs, including SCARs, take more time due to a long-lasting cell culture. Their protocols consist of more steps, which require handling instead of automated procedures. This can obviously result in a higher variability of results. Also, minor differences in experimental settings between laboratories, such as incubation time or sample storage conditions, may be a source of additional variability. Furthermore, different authors apply different methods to calculate the cut-off values in different assays, e.g., the stimulation index (cut-offs from 1.8 up to 4) [45,46], the mean of all the background samples plus two standard deviations [26,42], the mean of relative increase in control subjects plus 2–3 standard deviations [33,47], and receiver operator characteristic (ROC) curves [20,23,31]. Cut-off levels often rely on an average response to different drugs, which are tested together in the controls. However, a response may differ depending on a drug, e.g., radio-contrast media or β-lactams in LTT [3]. Thereby, the application of drug-specific thresholds in in vitro tests for DHR would be an optimal, albeit relatively rarely used, approach. To sum up, in the case of in vitro diagnostic assays for SCARs, the normal range in every single laboratory has to be well established and inter-laboratory comparisons should take into account the issues listed above.

Another concern relating to our results is the primary drug causality assessment, which is used as reference for investigated diagnostic laboratory tests. Different methods were applied in the identified studies from typical clinical history to very precise tools, such as the ALDEN algorithm. They are listed in the Section 2. Actually, they differ from one another in respect to probability of drug causality, which affects the results. If some patients with DHR tested with in vitro assays are not true positive cases, the obtained sensitivity is obviously biased downwards. Also, if two or more assays are performed in different patient groups with diverse percentages of true positive cases, the comparison between the assays is influenced by these differences.

### 3.4. Unmet Needs to Be Shown by the Study and Outlook for the Future

Undoubtedly, more studies have to be conducted. They should address the most promising methods and involve well-defined clinical entities (in respect to culprit drugs and clinical manifestations, e.g., allopurinol induced DRESS) and patients with a high probability of a causal relationship between the suspected drug and SCARs symptoms, especially patients from seldom investigated groups such as the pediatric population and AGEP cases.

Another challenge is managing potential external sources of variability (time span between symptoms and testing, time and conditions of samples storage, influence of drugs taken by patients on results), as well as internal sources of laboratory variability.

In view of the fact that investigated assays demonstrate low sensitivities in some patients, combining assays, which measure drug-specific responses in different end-points (e.g., granulysin, IFNg, granzyme B) may increase the overall sensitivity [20]. It seems to be a promising strategy in the most difficult cases, as long as it does not affect the specificity of the identification of the culprit drug.

Diagnostic laboratory methods in delayed DHR, especially SCARs, demand acquaintance with this field and practice and, therefore, at present, and most probably in the future, these methods are only performed in a limited number of specialized centers. However, an, at least partial, automation of laboratory techniques may contribute to the development of some in vitro methods, serving as a screening step in diagnostic process.

## 4. Methods

**Figure 1:**
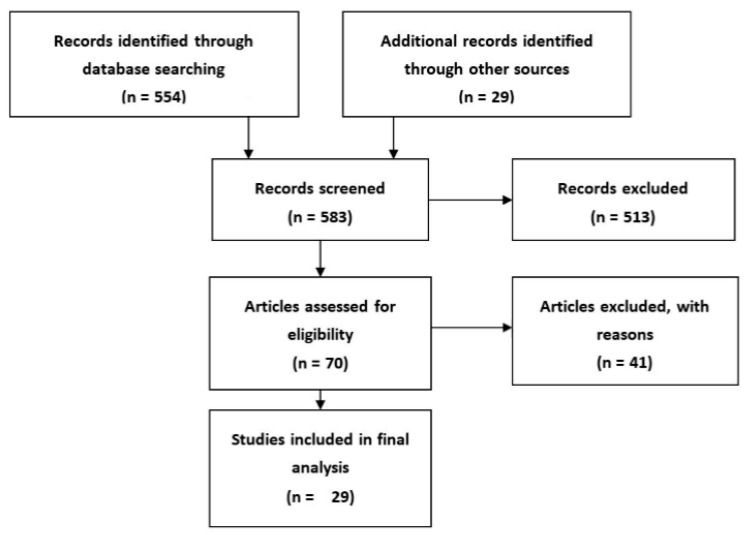
Identification, screening, and eligibility assessment of articles for analysis.

A comprehensive search of available English-language literature was carried out on PubMed. The search focused on studies describing or potentially describing diagnostic in vitro methods in severe cutaneous adverse drug reactions. The entry terms used were “in vitro”, “laboratory diagnosis”, “drug allergy”, “drug hypersensitivity”, “Stevens–Johnson Syndrome”, “SJS”, “Toxic Epidermal Necrolysis”, “Drug Reaction with Eosinophilia and Systemic Symptoms Syndrome”,” DRESS”, “Acute Generalized Exanthematous Pustulosis”, and “AGEP” and were combined using Boolean operators. The search strategy consisted of: set 1, “in vitro”, set 2, laboratory diagnosis; set 3, (“drug allergy” or “drug hypersensitivity” or “Stevens–Johnson Syndrome” or “Toxic Epidermal Necrolysis” or “SJS” or “Drug Reaction with Eosinophilia and Systemic Symptoms Syndrome” or “DRESS” or “Acute Generalized Exanthematous Pustulosis” or “AGEP”); and set 4, set 1 AND set 2 AND set 3. Searches were last updated on 7 April 2017. A total of 554 items were identified and assessed on the basis of their title and abstract or in full if no abstract was available. Forty-one were included in further analysis. An additional 29 references were identified from the bibliographies of the retrieved articles. In total, 29 articles from both groups met the eligibility criteria and were reviewed and evaluated in detail (Figure 1) [16,17,18,19,20,21,22,23,24,25,26,27,28,29,30,31,32,33,34,42,45,46,47,48,49,50,51,52,53].

A limitation that needs to be noted is the evaluation of the identified items by a single reviewer, however, the eligibility criteria adopted for a study inclusion were reasonably objective and evident. The studies considered for inclusion were conducted with the participation of patients with SCARs, who had clearly stated a diagnosis of SJS/TEN, DRESS, or AGEP (or whether a definite diagnosis could be drawn on the basis of the clinical data provided in an article) and underwent the unbiased selection to the study (i.e., consecutive individuals). We excluded case reports (due to possible bias for cases in favor of patients with positive results to diagnostic tests), studies with preselected patients (i.e., only individuals with positive LTT) and studies without the defined criteria for determining laboratory tests as positive or negative.

## 5. Conclusions

Patients, as well as many physicians, often expect a definitive answer to what is a causative drug in SCARs from in vitro tests. Such a clear-cut answer is only possible in some patients when the result of the test is clearly positive, which is limited by the sensitivity of the assay. Nevertheless, in vitro diagnostic tests possess a decisive influence on the management of a substantial part of SCARs patients.

At the moment, LTT is undoubtedly the best proven diagnostic assay in the clinical practice of SCARs. The next one: IFNg-ELISpot, is also quite well established. Further diagnostic assays, which are based mostly on distinct mechanisms of SCARs, may outdo the previous ones but they still need confirmation in larger groups of patients and in more research centers. To efficiently collect new evidence on the diagnostic accuracy of upcoming diagnostic assays, the studies should focus on well-defined patient groups. Data from pediatric populations and AGEP patients are scarce, which reflects difficulties in patients’ enrolment. Information about these groups is extrapolated from the adults and other SCARs, respectively.

## Figures and Tables

**Table 1:** Studies in which drug rash with eosinophilia and systemic symptoms (DRESS) patients were investigated.

Study	Endpoint (Activation Marker)	Read-Out System (Technique)	Drug	Patients			Controls
				N	Performed Tests	Positive Tests	N/Positive Tests
Karami, 2016	proliferation	LTT	aAEDs	9	9	5	24/1
Ye, 2016	proliferation	LTT	isoniazid	4	4	4	not reported
Cabanas, 2014	proliferation	LTT	piperacillin	8	8	8	not reported
Valeyrie-Allanore, 2014	proliferation	LTT	ND	12	12	4	20/0
Yun, 2013	proliferation	LTT	ALP/OXP ^(1)^	7	7	7	7/NA ^(2)^
Hanafusa, 2012	proliferation	LTT	aAEDs	3	3	2	not reported
Kano, 2007	proliferation	LTT	aAEDs	4	4	3	4/0
			mexiletine	1	1	1	
Kanny, 2005	proliferation	LTT	ICM	2	2	1	10/0
Naisbitt, 2003	proliferation	LTT	aAEDs	1	1	1	not reported
Ikeda, 1998	proliferation	BrdU FITC	Various ^(3)^	18	18	13	27/0
Houwerzijl, 1979	proliferation	LTT	aAEDs	7	7	7	10/0
Warrinoton, 1979	proliferation	LTT	isoniazid	8	8	6	19/0
Kato, 2016	proliferation	LTT	SMX/TRM	1	1	1	3/0 ^(4)^
	IFNg	ELISpot			1	1	3/0 ^(4)^
	proliferation	LTT	ALP/OXP	1	1	0	
	IFNg	ELISpot			1	0	
	proliferation	LTT	aAEDs	3	3	2	
	IFNg	ELISpot			3	0	
Haw, 2016	proliferation	LTT	ND	5	5	2	not reported
	IFNg	ELISpot			5	2	
	IL-4	ELISpot			5	5	
Polak, 2013	proliferation	LTT	ND	7	7	1	14/1 ^(5)^
	IFNg	ELISpot			7	5	14/1 ^(5)^
	IL-4	ELISpot			5	4	14/2 ^(5)^
Sachs, 2002	proliferation	LTT	aAEDs	2	2	2	15/0
	IFNg	ELISA			1	1	5/3
	IL-5	ELISA			2	2	15/0
	IL-10	ELISA			2	2	5/0
Chung, 2015	proliferation	LTT	ALP/OXP ^(1)^	3	3	1	not reported
	Grl	ELISA		7	7	6	34/3
Klaewsongkram, 2016	IFNg	ELISpot	ALP/OXP	13	13	9	21/1
Esser, 2012	IFNg	ELISpot	abacavir ^(6)^	12	12	5	17/0
Halevy, 2005	INFg	ELISA	various	3	3	2	22/not reported

^(1)^ only OXP induced positive response; ^(2)^ Controls not tested with LTT; ^(3)^ ceflazidim, ciprofloxacin, erythromycin, ethambutol, isoniazid, nifedipine, nilvadipine, ofloxacin, piperacillin, propafenone, propylthiouracil, pyrazinamide, rifampicin, ticlopidine; ^(4)^ It refers to the corresponding endpoint and read-out system; ^(5)^ Number of positive tests estimated from specificity given by authors, ^(6)^ Only HLA*57:01 negative individuals were analyzed. Abbreviations: N, number of the tested individuals (patients/ controls); aAEDs, aromatic antiepileptic drugs (phenobarbital, phenytoin, carbamazepine, lamotrigine); ALP/OXP, allopurinol/oxpurinol; BrdU FITC, bromodeoxyuridine fluorescein isothiocyanate assay; ELISA, enzyme-linked immunosorbent assay; ELISpot, enzyme-linked immunospot assay; Grl, granulysin; ICM, iodinated contrast media (ioxaglate, iohexol); LTT, lymphocyte transformation test; NA, not applicable; ND, not determined; SMX/TRM, sulfamethoxazole/trimethoprim.

**Table 2:** Sensitivity and specificity of different methods (wherever >5 DRESS patients were tested).

Method		Patients		Controls		Sensitivity	Specificity
		Performed Tests	Positive Tests	Performed Tests	Positive Tests		
proliferation	LTT/BrdU FITC	106	71	146	2	0.67	0.99
IFNg	ELISpot	42	22	55	2	0.52	0.96
IL-4	ELISpot	10	9	14	2	0.90 ^(1)^	0.86 ^(1)^
Grl	ELISA	7	6	34	3	0.86 ^(2)^	0.91 ^(2)^

^(1)^ based on data from a single center, ^(2)^ based on data from tests with a single drug (ALP/OXP), abbreviations: as in Table 1.

**Table 3:** Sensitivities corresponding with particular drugs (wherever >5 DRESS patients were tested with the same method).

Drug	Method		Patients		Sensitivity
			Performed Tests	Positive Tests	
aAEDs	proliferation	LTT	29	22	0.76
Abacavir	IFNg	ELISpot	12	5	0.42 ^(1)^
ALP/OXP	proliferation	LTT	11	8	0.73
	IFNg	ELISpot	14	9	0.64
	Grl	ELISA	7	6	0.86
isoniazid	proliferation	LTT	12	10	0.83
piperacillin	proliferation	LTT	8	8	1.00 ^(1)^

^(1)^ based on data from a single study, abbreviations: as in Table 1. Data for specificity not available (results for particular drugs not reported in control groups in most studies).

**Table 4:** Studies in which Stevens–Johnson syndrome/toxic epidermal necrolysis (SJS/TEN) patients were investigated.

Study	Endpoint (Activation Marker)	Read-Out System (Technique)	Drug	Patients			Controls
				N	Performed Tests	Positive Tests	N/Positive Tests
Karami, 2016	proliferation	LTT	aAEDs	11	11	7	24/1
Srinoulprasert, 2014	proliferation	LTT	sulfamethoxazole	1	1	0	3/0
	proliferation	LTT	aAEDs	1	1	1	
	proliferation	LTT	mefenamic acid	1	1	0	
Valeyrie-Allanore, 2014	proliferation	LTT	ND	22	22	7	20/0
Kano, 2007	proliferation	LTT	minocycline	1	1	0	4/0
			paracetamol	1	1	0	
Naisbitt, 2003	proliferation	LTT	aAEDs	2	2	1	not reported
Hari, 2001	proliferation	LTT	β-lactams	3	3	3	95/1
Roujeau, 1985	proliferation	LTT	Various ^(1)^	12	12	0	not reported
Kato, 2016	proliferation	LTT	celecoxib	1	1	0	3/0 ^(2)^
	IFNg	ELISpot			1	0	3/0 ^(2)^
	proliferation	LTT	ALP/OXP	1	1	0	
	IFNg	ELISpot			1	0	
	proliferation	LTT	aAEDs	1	1	0	
	IFNg	ELISpot			1	0	
Rozieres, 2009	proliferation	LTT	amoxicillin	1	1	1	11/0
	IFNg	ELISpot			1	1	11/0
Haw, 2016	proliferation	LTT	ND	4	4	2	not reported
	IFNg	ELISpot			4	4	
	IL-4	ELISpot			4	1	
Polak, 2013	proliferation	LTT	ND	9	9	5	14/1 ^(3)^
	IFNg	ELISpot			9	7	14/1 ^(3)^
	IL-4	ELISpot			8	4	14/2 ^(3)^
Klaewsongkram, 2016	IFNg	ELISpot	ALP/OXP	11	11	8	21/1
Halevy, 2008	IFNg	ELISA	paracetamol	1	1	1	11/4 ^(3)^
Chung, 2015	proliferation	LTT	ALP/OXP ^(4)^	2	2	1	not reported
	Grl	ELISA		7	7	6	34/3
Porebski, 2013 ^(5)^	proliferation	LTT	aAEDs	9	9	2	18/0 ^(2)^
	Grl ^(6)^	FACS			9	5	18/0 ^(2)^
	GrB	ELISpot			9	3	18/1 ^(2) (3)^
	IFNg	CBA			7	4	18/1 ^(2) (3)^
	proliferation	LTT	sulphonamides	4	4	1	
	Grl ^(6)^	FACS			4	1	
	GrB	ELISpot			4	1	
	IFNg	CBA			2	1	
	proliferation	LTT	ALP/OXP	1	1	0	
	Grl ^(6)^	FACS			1	1	
	GrB	ELISpot			1	1	
	IFNg	CBA			1	1	
	proliferation	LTT	mefenamic acid	1	1	1	
	Grl ^(6)^	FACS			1	1	
	GrB	ELISpot			1	0	
	IFNg	CBA			1	0	
Martin, 2010	IL-5 ^(7)^	FACS	aAEDs	1	1	1	10/0 ^(2)^
	IFNg ^(7)^				1	0	10/0 ^(2)^
	IL-10 ^(7)^				1	0	10/0 ^(2)^
	IL-5 ^(7)^	FACS	amoxicillin	1	1	1	
	IFNg ^(7)^				1	1	
	IL-10 ^(7)^				1	1	
	IL-5 ^(7)^	FACS	proguanil	1	1	1	
	IFNg ^(7)^				1	1	
	IL-10 ^(7)^				1	1	

^(1)^ oxyphenbutazone, cotrimoxazole, piroxicam, carbamazepine, fenbufen, flurbiprofen, noramidopyrine, sulfadiazine; ^(2)^ It refers to the corresponding endpoint and read-out system; ^(3)^ Number of positive tests estimated from specificity given by authors; ^(4)^ only OXP induced positive response; ^(5)^ only selected methods are shown; ^(6)^ in CD3^+^CD4^+^; ^(7)^ in CD3^+^CD4^+^ and CD3^+^CD8^+^. Abbreviations: N, number of the tested individuals (patients/ controls); aAEDs, aromatic antiepileptic drug (phenobarbital, phenytoin, carbamazepine, lamotrigine); ALP/OXP, allopurinol/oxpurinol; CBA, cytokine beads array; ELISA, enzyme-linked immunosorbent assay; ELISpot, enzyme-linked immunospot assay; FACS, fluorescence-activated cell sorting, applied in flow cytometry; GrB, granzyme B; Grl, granulysin; LTT, lymphocyte transformation test; ND, not determined.

**Table 5:** Sensitivity and specificity of different methods (wherever >5 SJS/TEN patients were tested).

Method		Patients		Controls		Sensitivity	Specificity
		Performed Tests	Positive Tests	Performed Tests	Positive Tests		
proliferation	LTT	95	35	192	3	0.37	0.98
IFNg	ELISpot	28	20	49	2	0.71	0.96
IL-4	ELISpot	12	5	14	2	0.42	0.86
Grl	ELISA	7	6	34	3	0.86^(1)^	0.91 ^(1)^
Grl in CD3^+^CD4^+^	FACS	15	8	18	0	0.53	1.00
GrB	ELISpot	15	5	18	1	0.33	0.94
IFNg	CBA	11	6	18	1	0.55	0.94

^(1)^ based on data from tests with a single drug (ALP/OXP), abbreviations: as in Table 1.

**Table 6:** Sensitivities corresponding with particular drugs (wherever ≥5 SJS/TEN patients were tested with the same method).

Drug	Method		Patients		Sensitivity
			Performed Tests	Performed Tests	
aAEDs	proliferation	LTT	29	14	0.48
aAEDs ^(1)^	Grl in CD3^+^CD4^+^	FACS	9	5	0.56
	GrB	ELISpot	9	3	0.33
	IFNg	CBA	7	4	0.57
ALP/OXP	proliferation	LTT	5	1	0.20
	IFNg	ELISpot	12	8	0.67
ALP/OXP ^(1)^	Grl	ELISA	7	6	0.86
Sulphonamides	proliferation	LTT	5	1	0.20

^(1)^ based on data from a single study, abbreviations: as in Table 1. Data for specificity not available (Results for particular drugs not reported in control groups in most studies).

**Table 7:** Studies in which acute generalized exanthematous pustulosis (AGEP) patients were investigated.

Study	Endpoint (Activation Marker)	Read-Out System (Technique)	Drug	Patients			Controls
				N	Performed Tests	Positive Tests	N/Positive Tests
Srinoulprasert, 2014	proliferation	LTT	amoxicillin	2	2	2	3/0
Gaspard, 2000	IFNg mRNA	RT-PCR	amoxicillin	2	2	2	11/4
	IL-4 mRNA	RT-PCR			2	0	11/0
Schmid, 2006	proliferation	LTT	ciprofloxacin	2	2	2	not reported

Abbreviations: lymphocyte transformation test (LTT); reverse transcription polymerase chain reaction (RT-PCR).

## Abbreviations

ADRs	Adverse drug reactions
aAEDs	Aromatic antiepileptic drugs
AGEP	Acute Generalized Exanthematous Pustulosis
ALDEN	Algorithm of drug causality for epidermal necrolysis
ALP	Allopurinol
BrdU FITC	Bromodeoxyuridine fluorescein isothiocyanate assay
CBA	Cytokine beads array
CD	Cluster of differentiation
CTLA4	Cytotoxic T-lymphocyte-associated protein 4
DHRs	Drug hypersensitivity reactions
DRESS	Drug Rash with Eosinophilia and Systemic Symptoms
ELISA	Enzyme-linked immunosorbent assay
ELISpot	Enzyme-linked immunospot assay
FACS	Fluorescence-activated cell sorting
GrB	Granzyme B
Grl	Granulysin
HLA	Human Leukocyte Antigens
ICM	Iodinated contrast media
IFNg	Interferon γ
IL	Interleukin
LTT	Lymphocyte transformation test
NA	Not applicable
ND	Not determined
OXP	Oxpurinol
PBMC	Peripheral blood mononuclear cells
PD-L1	Programmed cell death-ligand 1
RegiSCAR	Registry of severe cutaneous adverse drug reactions
ROC	Receiver operator characteristic curve
RT-PCR	Reverse transcription polymerase chain reaction
SCARs	Severe cutaneous adverse drug reactions
sFasL	Soluble factor-related apoptosis ligand
SJS	Stevens–Johnson Syndrome
SMX	Sulfamethoxazole
TCR	T-cell receptor
TEN	Toxic Epidermal Necrolysis
TNFa	Tumor necrosis factor α
TRM	Trimethoprim
